# Evaluation of Physicochemical Properties of Beetroot-Based Dietary Supplements

**DOI:** 10.3390/foods10081693

**Published:** 2021-07-22

**Authors:** Joanna Brzezińska, Adrian Szewczyk, Justyna Brzezicha, Magdalena Prokopowicz, Małgorzata Grembecka

**Affiliations:** 1 Department of Bromatology, Faculty of Pharmacy, Medical University of Gdańsk, Gen. J. Hallera Avenue 107, 80-416 Gdańsk, Poland; joanna.brzezinska@gumed.edu.pl (J.B.); justyna.brzezicha@gumed.edu.pl (J.B.); 2 Department of Physical Chemistry, Faculty of Pharmacy, Medical University of Gdańsk, Gen. J. Hallera Avenue 107, 80-416 Gdańsk, Poland; adrian.szewczyk@gumed.edu.pl (A.S.); magdalena.prokopowicz@gumed.edu.pl (M.P.)

**Keywords:** dietary supplement, average weight, disintegration time, friability, hardness, quality, beetroot

## Abstract

In the European Union, no specific requirements for the physicochemical parameters of dietary supplements have been established, contrary to the United States of America. This research aimed to assess the selected physical parameters of 31 commercially available beetroot-based dietary supplements in the form of tablets and capsules following the United States Pharmacopoeia (USP) guidelines and the Food and Drug Administration (FDA) recommendations. There was also estimated zinc and iron content by atomic absorption spectroscopy with flame detection. Results showed that nine products did not meet the USP requirements. Seven supplements needed more than 30 min to disintegrate. Two products in the form of tablets did not pass the friability test because of cracking. The hardness values varied significantly between manufacturers, demonstrating values from 59.1 to 455.8 N. The iron-enriched supplements differed significantly in iron content compared with the manufacturers’ declaration (84.91–140.69%). Inappropriate quality of dietary supplements, which may constitute a potential risk to consumers, can be related to the lack of specific regulations in Europe; hence, similar to the USA requirements should be considered in the European Union. The work emphasizes the need to better control the quality of dietary supplements before they are introduced to the European market.

## 1. Introduction

By definition [1], a dietary supplement is regulated as food and can only supplement a balanced diet; it cannot exhibit medicinal properties. The European Food Safety Authority (EFSA) is an international organization which role is to provide scientific advice and notices on existing and emerging risks associated with the food chain in Europe [2]. The major health-related risks and problems raised by dietary supplements of inappropriate quality are handled by the national authorities of member states. The Polish dietary supplements market is inspected by the Chief Sanitary Inspectorate (GIS). Moreover, the Polish dietary supplements market is monitored by the Office of Competition and Consumer Protection and The Trade Inspection Authority (IH) [3,4]. Medicinal products are assessed, supervised, and monitored by the European Medicines Agency at the European Union level. In Poland, a similar role is played by the Office for Registration of Medicinal Products and Medical Devices (URPL). In contrast, in the United States, one organization fulfills all of these roles. The Food and Drug Administration (FDA) in the USA is responsible for protecting and promoting public health through the control and supervision of food safety, dietary supplements, prescription and over-the-counter pharmaceutical drugs, and cosmetics [5]; this can facilitate the harmonization of requirements and improve market control. In conclusion, EFSA is the consultative body for countries in the European Union, while the FDA is a federal agency of the US Department of Health and Human Services.

The registration procedure of supplements on the Polish market is relatively easy and free of charge (after submission of appropriate notification, the distribution can start) [6]. As a result, a massive number of new products’ announcements are submitted to the GIS every year. For example, in 2020 there were 24,629 formulations reported to the products register covered by the notification of the first placing on the market [7].

The European and Polish legislation (unlike the American one) does not provide any requirements for the formulation, except that it should be dosable and cannot exert a pharmacological effect. The United States Pharmacopoeia (USP 43-NF 38) includes chapters <2040> and <2091> on testing of disintegration and dissolution, and weight variation of dietary supplements respectively [8,9]. Dietary supplements are produced applying the comparable to pharmaceutical products’ technology and occur in similar dosage forms. Therefore, any quality requirements regarding these forms and in vitro dissolution tests are required [8]. 

Recently, beetroot (*Beta vulgaris* L.) consumption has been increasing owing to its high nutritional value, significant content of nutrients and bioactive substances [10]. A dynamic interest in beetroot-based supplements is observed as the number of these products steadily increases on the market [7]. The root and its preserves exhibit antibacterial, anti-inflammatory, antipyretic, antiproliferative, choleretic, diuretic, anticancer, and anti-diabetic properties [11,12,13]. These beneficial effects are mainly associated with betalains (plant pigments that have a potent antioxidant and anti-inflammatory effect) and nitrates(V), which converted in the body to nitric oxide, cause a vasodilatory effect [14]. Although the beetroot contains only 0.76 mg of iron in 100 g [15], it may exert a mild hematopoietic effect [16,17]. Iron is characterized by a relatively low degree of absorption (2–10%), which results from its nonheme form and the reduction in absorption caused by fiber and oxalic acid. Vitamin C is considered a nonheme iron absorption promoter because it participates in the iron metabolism, forms complexes with iron or reduces ferric to ferrous iron [18,19,20]. One of the factors limiting the assimilability of iron might be the presence of zinc at a mass ratio of 1:2 (Zn:Fe) [21]. 

The health-promoting effects of beetroot, proven in clinical trials [22,23,24], are related to the appropriate dose of bioactive substances delivered to the body, their release from the plant matrix, digestion, assimilation, and metabolism. Hence, it is related both to the quality of the product (including the substitute used in the supplement production) and its form. The individual formulation process factors exhibit a direct impact on the physical parameters of the final formation (hardness, abrasion, disintegration rate). They should also enable the effective release of the active substances from the dosage form. The uniformity of the weight of the dosage units is responsible for the reproducibility of the effect.

European legislation recommends manufacturers to perform the necessary analyses of supplement ingredients and finished products before placing them on the market [2,25,26]. Dietary supplements should be properly labeled and safe for the consumer, which is the responsibility of the producer. The manufacturer should also follow the guidelines that define the limits of chemical and biological contamination. The safety of the selected products is directly related to their quality. The quality of the dietary supplement concerns the physicochemical parameters of the finished form, the content of ingredients in accordance with the manufacturer’s declaration, the appropriate product labeling, and their effectiveness. Possible contamination above the permissible limits or adulteration reduces supplements’ quality and safety of use. For example, microbial contamination can reduce the stability and quality of the finished product and pose an immediate pathogenic risk. The presence of undeclared or unauthorized substances is associated with a significant danger, which may result in an unknown effect on the body and even permanent health damage [27,28,29]. Some products do not contain sufficient or declared amounts of the active ingredient(s), or might be contaminated with harmful constituents, or active substances are not released efficiently from the dosage form [29,30]. Furthermore, complex products (consisting of many ingredients) in tablets of relatively large size and weight are present on the market. Thus, problems with swallowing, a risk of retention, and sticking to the gastric mucosa can appear in consumers [31]. Additionally, in the case of beetroot-based dietary supplements, they may also induce hypotension (in people with low blood pressure or due to synergistic interaction with antihypertensive drugs) or exacerbate gout and kidney stones symptoms (owing to the content of oxalic acid) [32]. The state of plant-based dietary supplements is unregulated, as there are no requirements for products standardization in terms of specific substances or guidelines related to the formulation or the content of additional substances in a dosage unit. 

The research aimed to investigate the selected physical parameters (average weight, disintegration time, friability, hardness, size, and shape) of commercially available beetroot-based dietary supplements, as well as zinc, and iron content. These two metals’ content was evaluated because iron amount was declared on the label by the manufacturer while zinc potentially interacts with this element. The analyses of physical parameters were performed following the USP requirements for dietary supplements and tablet form. The obtained results were compared with manufacturers’ declarations and the USP guidelines. There are no recommendations in Europe for the form of dietary supplements that could be referred to when verifying the quality of the product available on the market. Therefore, the aim of the study was to investigate the quality of beetroot-based dietary supplements available on the Polish market in terms of their physicochemical characteristics. The obtained results should verify whether there is a need to introduce to the European market regulations concerning dietary supplements’ physical parameters similar to the USP guidelines.

## 2. Materials and Methods

Thirty-one commercially available supplements (twenty-three different manufacturers) made of beetroot or beetroot preserves were obtained from various drugstores or online shops on the Polish market. The full characteristic of the analyzed products (marked from A to Y) is presented in Table S1, which was prepared based on the information available on the supplement packaging. The graph with experimental design is depicted in Supplementary Material as Figure S1. The selection criterion was the availability of products during the 2-month collection period (October–November 2020) and the form for which physicochemical parameters can be determined according to the USP and FDA [5]. The analyzed group consisted of seventeen supplements in capsules (A–L) and fourteen formulations in the form of uncoated tablets (M–Y). The products were manufactured in European countries, as well as in the USA (Table S1). During the experiment period, eight products were purchased twice to assess the variability of parameters between series. As can be seen in Table S1, the products marked with identical letters and numbers 1 or 2 (e.g., A1 and A2) were manufactured by the same producer and sold under the same trade name. The investigated dietary supplements contained different beetroot preserves, i.e., dried juice, dried extracts, and lyophilisate. Eight supplements were enriched in iron compounds. 

### 2.1. Average Weight

All investigated dietary supplements were subjected to the weight variation test described in the USP 43-NF 38 guideline; <2091> chapter “Weight variation of dietary supplements” [9]. The mass of 20 individual tablets and capsules was weighted using an analytical balance ±0.0001 g (Semi-micro balance TS2215Di, VWR, Leuven, Belgium). Based on pharmacopeia [8], the following limits were set for tablets: not more than two of the investigated tablets could differ from the average weight of a sample by more than ±5% and no tablet could differ by more than ±10%. The net weights of capsules were calculated by subtracting the weights of the shells from the respective gross weights. The average net content from the sum of the individual net weights was determined. The requirements were fulfilled unless more than two of the differences between each net content and average net content were greater than 10% and, in no case, the difference was greater than 25%.

### 2.2. Friability Test

To conduct friability testing, the USP 43-NF 38 guideline; <1216> chapter “Tablet friability” [33] was implemented. For tablets with a unit weight ≤ of 650 mg, a sample of 12 whole tablets corresponding to approx. 6.5 g was used. For tablets with a unit weight > 650 mg, 10 whole tablets were tested. Dust-free tablets were placed in a pharmacopeia friabilator (Friability tester TAR 10, Erweka, Warsaw, Poland) and rotated for 4 min with 25 rpm. The friability (F) was calculated using Equation (1):

(1) $$F=\frac{w_{1}-w_{2}}{w_{1}}\cdot 100\%$$

where *w*_1_ and *w*_2_ are the masses (g) of the tablets before and after the test, respectively. Tablets, for which friability was ≤1%, met the pharmacopeia requirements. Cleaved, cracked, or broken tablets failed the test. 

### 2.3. Hardness Test

The hardness of tablets, defined as the breaking force that causes tablets fracture, was determined using a hardness tester (Tablet hardness tester TBH 125, Erweka, Warsaw, Poland) in agreement with the USP 43-NF 38 guideline; <1217> chapter “Tablet breaking force” [34]. The measurement was carried out for 10 tablets of each dietary supplement and expressed as an average value ± standard deviation. 

### 2.4. Disintegration Time

The disintegration time of investigated dietary supplements was carried out based on the USP 43-NF 38 <2040> chapter “Disintegration and dissolution of dietary supplements” [8]. For this purpose, pharmacopeia disintegration apparatus was used (Disintegration tester ZT 320, Erweka, Warsaw, Poland), raising and lowering the basket in the immersion fluid at a constant frequency rate of 29–32 cycles per min. An amount of 0.1 M HCl (37 ± 2 °C) was used as an immersion fluid to simulate the pH of the stomach. Six individual tablets or capsules of each dietary supplement were investigated. The following pharmacopeia limit was set: after 30 min of the test, all of the investigated tablets and capsules should be completely disintegrated.

### 2.5. Analysis of Size and Shape

**Figure 1:**
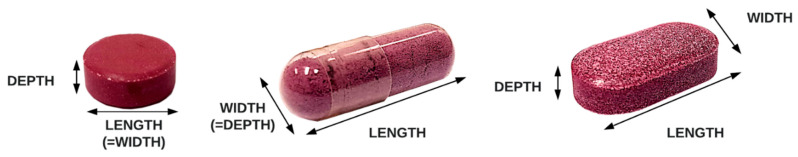
Indices of supplements formulations size.

Due to the lack of guidelines concerning the size and shape analysis of the solid dosage form of dietary supplements, Overgaard et al. [35] approach was applied. Five specific categories were used to properly evaluate the shape of the units: cylindrical capsules, arched circular tablets, flat circular tablets, arched oblong tablets, and arched oval tablets [35]. Ten dosage units of each product were measured with a caliper (Vis, Warsaw, Poland, measuring range 0–15 cm, resolution up to 0.05 mm). Length and width (=depth) were measured for capsules; length (=width) and depth for circular tablets; and width, length, and depth for oval or oblong tablets (Figure 1). Tablets whose length = width were classified as circular. If length > width, tablets were classified as oblong (the same width in every point of tablet) or oval (the smaller width the further from the middle of the tablet). Tablets were classified as flat when the top and bottom surfaces were flat, or arched when they were convex. Industry guidelines published by the FDA regarding the size and shape of capsules and tablets were also used to evaluate the analyzed supplements [5].

### 2.6. Preparation of Samples to Flame Atomic Absorption Spectroscopy (FAAS) Analysis

All the analyzed dietary supplements’ samples were weighted and homogenized using mortar. Next, 0.3 g (±0.0001 g) portions of samples were transferred to Teflon bombs. 8 mL 65% HNO_3_ (Suprapur Merck^®^, Darmstadt, Germany) and 2 mL 30% H_2_O_2_ (Suprapur Merck^®^, Darmstadt, Germany) were added to the samples, which were digested using ETHOS One Milestone microwave system. The applied program was as follows: 0–20 min, 1500 W, 180 °C; 10 min, 1500 W, 180 °C. After the mineralization process, the solution was transferred to 25 mL flasks with ultrapure water (18 MΩ cm^−1^) from the Mili-Q system (Millipore, MA, USA). Each sample was prepared in triplicate.

### 2.7. Analysis of Iron and Zinc

The concentrations of iron (Fe) and zinc (Zn) were determined by atomic absorption spectrometry with an air-acetylene flame (FAAS) with deuterium background correction [36]. The analysis was performed in triplicate using Thermo Scientific i3000 (Waltham, MA, USA). 

The limit of detection (LOD) and limit of quantification (LOQ) of the applied method were determined following the formula proposed by Konieczka and Namieśnik [37], i.e., LOD = average + 3 SD and LOQ = 3 ∗ LOD. The LOD of the iron determination method was 0.053 μg/mL and LOQ 0.158 μg/mL. The LOD of the zinc determination method was 0.010 μg/mL and LOQ 0.030 μg/mL.

To fully validate the method the certified reference material Oriental Basma Tobacco Leaves (INCT-OBTL-5) was analyzed according to the same procedure as the dietary supplements samples. The recovery and precision obtained for the tested material amounted to 107% and 0.63% for Fe, 107% and 4.31% for Zn, respectively.

### 2.8. Statistical Analysis

Each sample was tested in triplicate. Collected data are presented as the average ± standard deviation (SD). The Shapiro–Wilk test was applied to check the normality of analyzed results; statistical significance was defined as *p* < 0.05. Differences between the manufacturers’ declaration and empirically determined results were analyzed using a two-tailed unpaired Student’s *t*-test. Differences between batches of the same product were estimated by Mann–Whitney U test (for non-normally distributed data)*;* statistical significance was defined as *p* < 0.05. Correlation between parameters was evaluated using a Spearman’s rank correlation coefficient. All analyses were performed using Statistica 13.3 (TIBCO Software Inc., Palo Alto, CA, USA).

## 3. Results and Discussion

**Figure 2:**
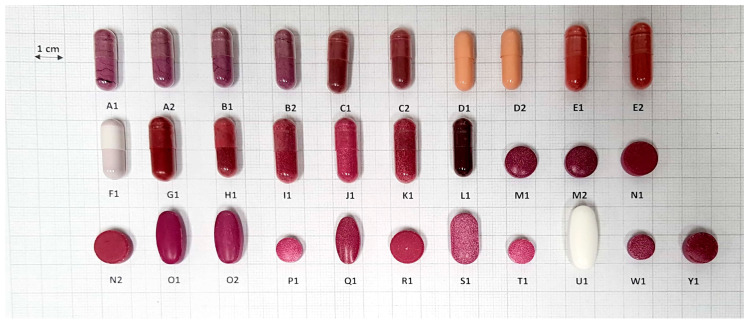
Variation of sizes and shapes of the analyzed dietary supplements.

The physicochemical quality control analyses together with iron and zinc content determinations were performed for beetroot-containing capsules and tablets. All products (coded A–Y, Table 1 and Table 2) were tested for weight variation and disintegration. Uncoated tablets (M–Y) were checked for friability and hardness according to the relevant USP criteria [8,9,33,34]. All products (A–Y) were also analyzed for the iron and zinc content using FAAS. Eight of them (GL1, GL2, HE1, HE2, HF1, HF2) were enriched in iron compounds by manufacturers (Table S1), and their content was specified on the package. The investigated dietary supplements, obtained from various producers, contained different beetroot preserves such as dried juice, dried extracts, and lyophilizate and were characterized by diverse shapes and sizes (Figure 2).

### 3.1. Weight Variation of the Dosage Form

The average weight of the investigated capsules and tablets, together with the weight variation, are presented in Table 1 and Table 2, respectively. Both capsules and tablets were characterized by wide weight values in the range of 379.35–823.55 mg for capsules and 352.33–1528.94 mg for tablets. This range of weight values can be partially explained by the fact that the dietary supplements were purchased from various manufacturers; thus, exhibited a large variety of shapes and sizes (Figure 2). All the analyzed tablets passed the pharmacopeial weight change test (Table 2). However, two products (A2, L1), in the form of capsules, failed the pharmacopeial requirements, because in both cases the net weight of three samples differed from the average net weight more than 10% (Table 1). Nonetheless, >90% of the tested supplements demonstrated suitable uniformity of weight according to the USP. 

Among the investigated dietary supplements, only 23 products were characterized by the declared weight by the manufacturer. There were found statistically significant differences between the average weight and the declared weight for 17 of the analyzed products. In particular, for three of them (A2, B1, B2) the average weight was significantly lower, while for 14 (C1, D1, E1, E2, L1, N1, N2, O1, O2, Q1, S1, T1, U1, W1) higher than declared (t-Student test, *p* < 0.05). No statistically significant differences in weight values for two batches of the same product were noticed (Table 1, Mann–Whitney U test, *p* > 0.05). Similarly, no differences were found for tablets (Table 2, Mann–Whitney U test, *p* > 0.05) except for D1 and D2 (Mann–Whitney U test, *p* = 0.02) as well as N1 and N2 (Mann–Whitney U test, *p* = 0.00). 

It is well-known that the uniformity of the weight of the individual dosage units guarantees the uniformity of the dosage of the product. This parameter is particularly important to provide the same dose of active substances during each administration, therefore, to ensure the repeatable pro-health effect [38]. The proven uniformity of weight of the investigated dietary supplements indicates their repeatable production. However, it should be noted that O1, O2, S1, and U1 supplements in the form of tablets were characterized by a dosage unit weight greater than 1 g, which may impede the simple administration of a product by the consumers [39]. Large tablets with an average weight higher than 1 g might be difficult to swallow and increase the potential risk of choking. That might be of crucial importance for geriatric patients [40].

### 3.2. Hardness and Friability Tests

The hardness and friability tests were performed for tablets (Table 2) to evaluate the mechanical strength and susceptibility to breakage, due to falls or friction [33,34]. Similar to weight variation results, the hardness values differed significantly between manufacturers demonstrating values from 59.1 to 455.8 N (Table 2). Nonetheless, various batches of tablets obtained from the same manufacturer did not show significant differences in hardness values and were characterized by similar friability values (Mann–Whitney U test, *p* > 0.05). However, both batches of the dietary supplement Q (Table 2) did not pass the pharmacopeial friability test, because of tablets cracking during the test. Hardness values of tablets obtained from different manufacturers indicated a low correlation coefficient with the weight of the dosage unit (Spearman’s ρ = 0.09) as well as with the disintegration time (Spearman’s ρ = 0.52). Thus, tablets with higher average weights did not display increased hardness values. 

### 3.3. Disintegration Test 

The results of the disintegration time of capsules and tablets are presented in Table 1 and Table 2, respectively. The dietary supplements in the form of capsules disintegrated relatively faster (4–31 min) than tablets (13 to >60 min). The average disintegration time did not vary substantially between different batches of one manufacturer, excluding dietary supplement Q for which it differed two-fold (23 and 44 min for Q1 and Q2). Seven of the dietary supplements (six in the form of tablets, one in the form capsules) that constituted >20% of all investigated products, did not pass the pharmacopeia requirements of disintegration time. After 30 min of the disintegration test, more than two dosage units did not disintegrate completely in each of these seven products. In extreme cases, for products in the form of tablets, more than 40 min (products O2, S1, U1) or 1 h (product Y1) were required to observe the disintegration. 

An adequate tablet strength should provide fracture toughness to permit dosing and, at the same time, adequate disintegration upon digestion. Unfortunately, inappropriate manufacturing of dosage form (e.g., high compression force, unsuitable excipients) may inhibit both the disintegration of dosage form and dissolution of active substances [38]. In such a case, the digestive processes, after in vivo administration of the product, may not be sufficient to provide the liberation of the components for absorption. Based on the obtained results, there is a potential risk that the dietary supplements O2, P1, S1, U1, W1, and Y1 will not completely disintegrate after consumption, which may result in the failure of both the total dose release and the absorption. Moreover, considering the administration of tablets by patients, it might be impeded or even impossible to swallow the tablets with weight exceeding 1 g, and which are too tough to be crushed or dissolved in a glass of water. Positive correlations between average weight, hardness, and disintegration time for four formulations (O1, O2, S1, and U1) were observed (Spearman’s ρ = 0.8). Therefore, higher average weight of these dietary supplements was associated with both higher hardness and longer disintegration time.

The lack of formulation disintegration may also result from the interaction of the raw materials used with the capsule shell material. Gusev et al. [41] found that some supplements’ ingredients (such as epigallocatechin gallate) may require more biologically relevant destructive forces to disintegrate and the USP protocols should consider different botanicals. Other research data also indicate poor quality and formulation issues with dietary supplements assessed by the USP requirements [38,42].

### 3.4. Analysis of Size and Shape

The results of size and shape measurements are presented in Table 3. As shown in Figure 2, the analyzed dietary supplements were characterized by a broad variety of shapes and sizes. The analyzed formulations were categorized [35] as cylindrical capsules (17 products), arched circular tablets (six products), flat circular tablets (three products), arched oblong tablets (three products), and arched oval tablets (two products). 

The FDA recommends that “the largest dimension of a tablet or capsule should not exceed 22 mm and that capsules should not exceed a standard 00 size” [43]. In Europe, capsule 00 size capacity is 0.95 mL, length is 23.3 ± 0.3 mm, and diameter is 8.56 mm. Patients reported swallowing difficulties related to tablet diameter greater than 8 mm [44,45]. It has been proven that adverse effects such as pain, gagging, choking, and aspiration are associated with ingestion problems in the oropharyngeal phase of swallowing and are increasingly common with greater formulation sizes (>8 mm diameter) [46,47]. Furthermore, swallowing difficulties of products may result in patient noncompliance with regimens. The acceptability of the size of the formulation is also related to the dosage form. The ideal form indicated by patients is a small, strongly arched, and coated tablet. Generally, patients preferred capsules over tablets and coated tablets over uncoated [35].

One analyzed formulation did not meet the FDA recommendations (U1; Table 3). because it had a diameter larger than 22 mm. The eleven formulations (capsules) were potentially convenient to ingest for patients, but A2 and L1 did not pass quality tests. However, numerous scientific reports [40,44,45,48] indicate that tablets exceeding 8 mm (in any dimension) are not preferred by patients due to discomfort during ingestion. According to this criterion, 13 supplements might be inconvenient for patients to swallow. Moreover, Kabeya et al. [49] designated the index of tablet/capsule size, summing up the length, width, and depth values of the evaluated formulations, and the result above 21 mm was associated with negative feelings of patients related to ingesting difficulties. According to these findings, each of the analyzed beetroot-based dietary supplements may be potentially problematic to swallow. Nonetheless, this research [49] has a limitation because it was conducted on the Japanese population, so it cannot be directly transferred to other populations. Among 31 tablets and capsules analyzed in our study, ranging in size (length or width) from 6.70 to 23.55 mm, twenty formulations were ≥8 mm, and consequently their swallowing might be inconvenient for elderly people, especially patients suffering from dysphagia [48]. This highlights the need to develop formulations with the best adapted characteristics to reach optimal acceptability in targeted users and to put appropriate information on the package. 

### 3.5. Iron and Zinc Content

Iron is a common ingredient of dietary supplements, usually applied in the form of ferric and ferrous salts (such as iron(II) gluconate, iron(II) fumarate, iron(II) sulfate, iron(III) citrate, and iron(III) sulfate). Ferrous ion is characterized by a higher bioavailability than ferric ion because of a higher solubility [50]. When analyzing the product’s label, it is worth noting that individual salts contain varying amounts of elemental iron, for example, iron(II) fumarate consists of 33% elemental iron by weight, while iron(II) gluconate of 12% elemental iron. Supplementation with high doses of iron (over 45 mg/day) may result in gastrointestinal side effects, such as nausea, constipation, and diarrhea, or even more severe results caused by an accumulation of iron in the organism (cardiovascular, nervous system, kidneys, and liver disorders) [51].

**Figure 3:**
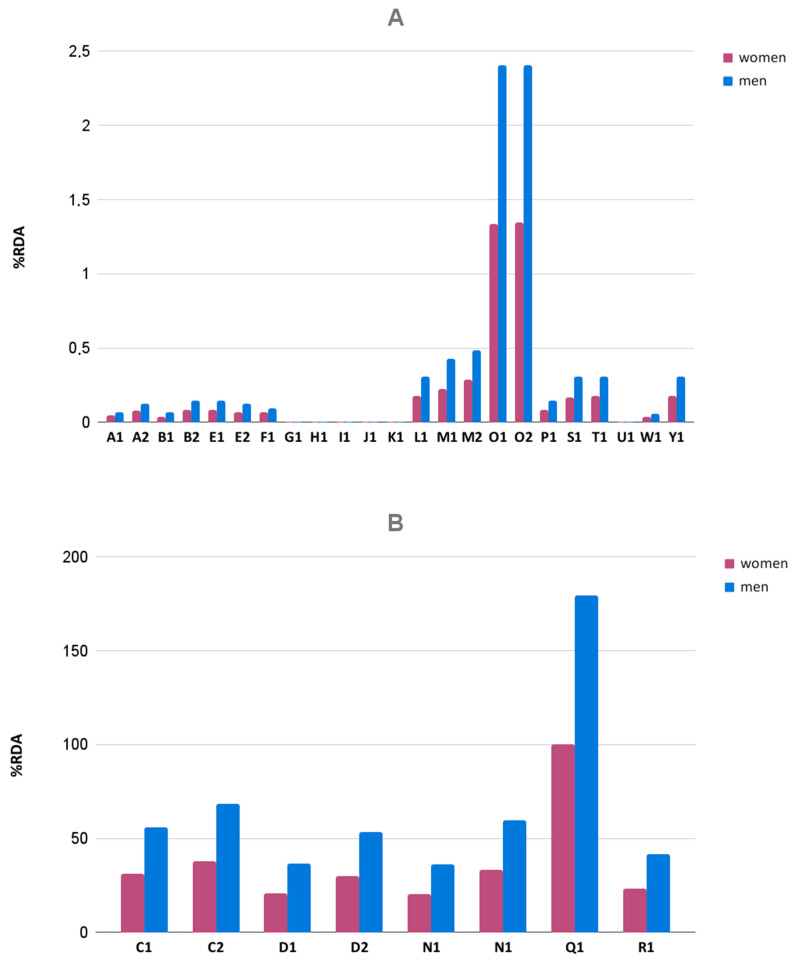

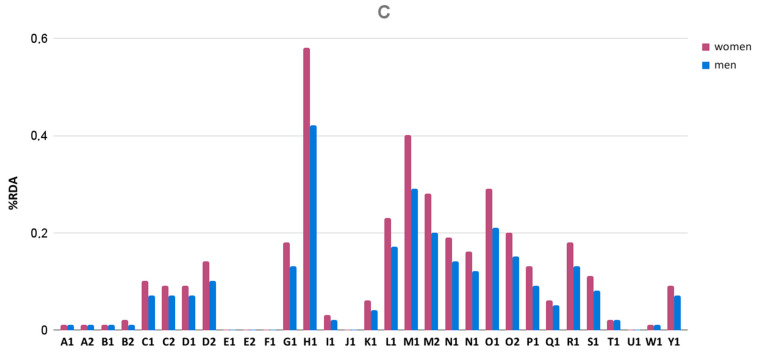
(**A**) Realization of RDA for iron by daily consumption of the analyzed unenriched dietary supplements. (**B**) Realization of RDA for iron by daily consumption of the analyzed enriched dietary supplements. (**C**) Realization of RDA for zinc by daily consumption of the analyzed dietary supplements.

Eight dietary supplements (C1, C2, D1, D2, N1, N2, Q1, R1) were enriched in iron compounds (iron(II) gluconate or iron(II) fumarate. Five of them (C2, D1, N1, N2, Q1, R1; Table S2) displayed statistically significant differences in this microelement content in comparison with manufacturers’ declarations (*t*-Student test, *p* < 0.05). Iron levels ranged between 84.91 and 140.69% of producers’ declarations. Moreover, all the analyzed pairs of products displayed statistically significant differences between batches (Mann–Whitney U test, C1 and C2: *p* = 0.01; D1 and D2: *p* = 0.01; N1 and N2: *p* = 0.01). Based on the determined iron content and the recommended intake of the supplement by the manufacturer (Table S2), the daily amount of iron supplied was estimated. This amount was evaluated according to Polish Recommended Dietary Allowance (RDA) guidelines, which are 18 mg for women and 10 mg for men between 19 and 50 years old [52]. Simultaneously, the RDA guidelines by the National Institutes of Health (NIH) are 18 mg of iron for women and 8 mg for men, between 18 and 50 years old and nonvegetarians [53]. Enriched supplements allow the realization of the Polish RDA for iron within the range of 19.81 and 99.56% for women and 35.67 to 179.2% for men, taking into account the dosage recommended by the manufacturer (Figure 3A–C). Unenriched products contained lower iron amounts ranging between 0.00–0.04 mg of iron/dosage unit, which implies 0.00–1.34% of RDA for women and 0.00–2.4% of RDA for men. In the case of zinc, the RDA values according to Polish and American guidelines are the same [52,54] amounting to 8 mg for women and 11 mg for men, above 19 years old [52]. The analyzed supplements provided from 0.0 to 0.58% of RDA for zinc for women and from 0.0 to 0.42% of RDA for men (Figure 3C). Based on the obtained results, it was found that dietary supplements enriched with iron compounds can be valuable sources of this element, provided that the appropriate physicochemical quality of the formulation is ensured. Moreover, the iron content in 56% of the unenriched supplements was higher than in dried beetroot (0.76 mg/100 g dry weight) [15]. In the case of zinc, 71% of all supplements had a higher content of this element than the dried beetroot (0.29 mg/100 g dry weight) [15].

It is considered that the combined supplementation of zinc and iron may limit the absorption of each other at a mass ratio of 1:2 (Zn:Fe) [21]. Zinc and iron mass ratios in dosage units were calculated based on the determined content (Table S2). Computed ratios show that zinc in iron-enriched supplements will not affect iron absorption, while in unenriched supplements both elements are at levels where there will be no interaction. 

Iron deficiency is one of the major contributors to the global burden of disease. Children, premenopausal women, and people in low and middle-income countries are particularly affected. One of the numerous consequences is anemia, and in most cases, oral iron therapy is the first line of treatment [55]. Consumption of iron-fortified foods is considered an effective method of hemoglobin and serum ferritin level improvement with a reduced risk of remaining anemic and iron-deficient [56]. Furthermore, the cumulative effect of vitamins and iron exerts a protective role in decreasing all-cause mortality and cardiovascular diseases. This effect is observed in relation to higher dietary iron intake [57]. 

## 4. Conclusions

The quality of the dietary supplement concerns the physicochemical parameters of the final form, the content of ingredients in accordance with the manufacturer’s declaration, the appropriate product labeling, and their effectiveness. This work aimed to assess the physicochemical parameters of dietary supplements available in Europe because there is a lack of such data. It was carried out in accordance with the USP guidelines as there are no adequate European guidelines for the formulation of the product, which may result in the marketing of products of unknown quality. The results confirmed that there is a need to control beetroot-based dietary supplements, as they show considerable variation in physical parameters even between batches of products obtained from the same producer. Some of them may pose risks (such as difficulties with swallowing, choking, gastric sticking, and gastric erosions or ulcers) to the consumer due to the size and shape of the unit dose, non-degradation, and high hardness or friability. In addition, it can be concluded from the producer’s declaration that most products contain a negligible amount of processed beetroot (usually less than 5 g of fresh vegetables). Only supplements enriched with iron deserve attention, as they can be important sources of this ingredient compared to other products. The effectiveness of dietary supplements is inherently connected with the content of bioactive substances. However, this study focused on the assessment of physicochemical parameters and the content of iron and zinc in the tested products. Determination of the other beetroot phytochemicals content will be the subject of further research.

In summary, similar to the USP requirements should be considered on the European market of dietary supplements, and control of the formulation quality should be provided to ensure safety for consumers. Moreover, due to the increasing number of adulterated dietary supplements, there is a need for constant monitoring of their chemical composition and safety. Possible contamination above the permissible limits or adulteration translates into an increased health hazard for the consumer. Therefore, stricter controls before placing them on the market should be considered.

## Figures and Tables

**Table 1:** Physical parameters of supplements in capsule form.

Code	Weight Uniformity							Disintegration Time	
	Declared Weight of the Dosage Unit (mg)	Average Gross Weight (mg)	Average Net Weight ± SD (mg) ^1^	Min% ^2^	Max% ^3^	Pharmacopeia Criteria	Ratio of Average Weight to Declared Weight (%)	Disintegration Time (min)	Pharmacopeia Criteria
A1	500	496.75 ± 15.25	397.15 ± 13.81	−5.07	+6.26	passed	99.35	5:00	passed
A2	500	486.15 ± 22.5	392.95 ± 25.75	−14.26	16.02	failed	97.23	4:30	passed
B1	500	490.05 ± 14.59	403.45 ± 15.32	−9.53	+5.59	passed	98.01	5:00	passed
B2	500	492.55 ± 11.88	399.75 ± 10.93	−5.82	+5.19	passed	98.51	4:00	passed
C1	596	608.05 ± 12.48	512.9 ± 12.37	−5.98	+5.25	passed	102.02	5:30	passed
C2	596	599.7 ± 18.17	502.95 ± 18.18	−9.04	+7.81	passed	100.62	6:00	passed
D1	376	388.1 ± 9.06	311.65 ± 7.86	−5.05	+4.11	passed	103.22	8:00	passed
D2	376	379.35 ± 13.9	302.65 ± 13.65	−9.95	+6.95	passed	100.89	6:30	passed
E1	690	735.3 ± 26.69	599.8 ± 26.66	−6.84	+4.72	passed	106.57	9:00	passed
E2	690	751.65 ± 25.94	614.45 ± 26.64	−6.34	+7.10	passed	108.93	9:00	passed
F1	NA*	601.96 ± 11.96	506.59 ± 11.58	−6.26	+3.62	passed	ND*	12:00	passed
G1	NA*	832.55 ± 12.8	713.54 ± 13.62	−3.77	+2.90	passed	ND*	14:00	passed
H1	NA*	634.80 ± 10.65	539.90 ± 11.24	−3.82	+3.61	passed	ND*	11:00	passed
I1	NA*	750.14 ± 24.84	625.28 ± 23.45	−8.14	+5.43	passed	ND*	17:00	passed
J1	NA*	719.70 ± 19.12	584.43 ± 18.69	−11.93	+3.08	passed	ND*	11:00	passed
K1	NA*	748.52 ± 14.87	628.28 ± 16.07	−4.37	+4.60	passed	ND*	13:00	passed
L1	300	382.65 ± 18.82	286.11 ± 18.25	−10.04	+14.92	failed	127.55	31:00	failed

^1^ weight of capsule filling without capsule shell. ^2^ % difference between average net weight and the lowest net weight of investigated samples. ^3^ % difference between average net weight and the highest net weight of investigated samples. ND*—no data; value cannot be calculated due to lack of data from the manufacturer. NA*—not available; no specification on the package.

**Table 2:** Physical parameters of supplements in tablet form.

	Weight Uniformity						Disintegration Time		Friability		Hardness		
Code	Declared Weight of the Dosage Unit (mg)	Average Weight ± SD (mg)	Min% ^1^	Max% ^2^	Pharmacopeia Criteria	Ratio of Average Weight to Declared Weight (%)	Disintegration Time (min)	Pharmacopeia Criteria	Value (%)	Pharmacopeia Criteria	Value ± SD (N)	Min (N)	Max (N)
M1	550	547.65 ± 11.21	−4.68	+2.80	passed	99.57	17:30	passed	0.24	passed	204.50 ± 12.28	175	224
M2	550	553.20 ± 18.19	−7.81	+3.40	passed	100.58	13:30	passed	0.27	passed	155.70 ± 12.37	138	172
N1	650	671.15 ± 3.73	−0.92	+0.87	passed	103.25	13:00	passed	0.06	passed	59.10 ± 14.27	39	82
N2	650	659.60 ± 5.43	−1.76	+1.27	passed	101.48	15:50	passed	0.18	passed	164.40 ± 45.05	55	211
O1	925	1033.45 ± 8.49	−1.01	+2.18	passed	111.72	22:50	passed	0.97	failed	119.80 ± 9.53	105	136
O2	925	1036.00 ± 8.30	−1.25	+1.74	passed	112.00	44:00	failed	0.88	failed	121.50 ± 14.57	100	148
P1	350	352.33 ± 13.37	−11.88	+5.17	passed	100.67	30:40	failed	0.16	passed	180.5 ± 77.75	26	322
Q1	630	725.28 ± 3.53	−0.89	+0.66	passed	115.12	16:50	passed	0.21	passed	125.3 ± 3.74	118	132
R1	650	653.15 ± 7.40	−2.77	+2.00	passed	100.48	15:10	passed	0.25	passed	198.7 ± 19.93	169	235
S1	NA*	1063.88 ± 31.54	−9.88	+3.27	passed	ND*	40:00	failed	0.06	passed	455.8 ± 31.67	386	488
T1	350	355.28 ± 6.05	−2.77	+3.51	passed	101.51	20:30	passed	0.22	passed	104 ± 16.29	84	138
U1	1460	1528.94 ± 29.93	−2.44	+7.50	passed	104.72	48:00	failed	0.07	passed	241.6 ± 74.74	21	286
W1	350	353.88 ± 3.96	−1.71	+1.80	passed	101.11	37:20	failed	0.15	passed	420.5 ± 167	381	440
Y1	NA*	812.42 ± 7.70	−1.54	+2.22	passed	ND*	>1 h	failed	0.44	passed	216.5 ± 15.04	193	250

^1^ % difference between average weight and the lowest weight of investigated samples. ^2^ % difference between average weight and the highest weight of investigated samples. ND*—no data; value cannot be calculated due to lack of data from the manufacturer. NA*—not available; no specification on the package

**Table 3:** Analysis of shape and dimensions of formulations.

Form	Product	Shape	Shield/ Coating ^1^	Width ± SD (mm)	Length ± SD (mm)	Depth ± SD (mm)	W + L + D (mm) ^4^	FDA Recommendation ^5^
CAPSULES	A1	cylindrical capsule	gelatin	7.44 ± 0.06	21.49 ± 0.16	7.44 ± 0.06	36.36	acceptable
	A2	cylindrical capsule	gelatin	7.40 ± 0.06	21.46 ± 0.18	7.40 ± 0.06	36.26	
	B1	cylindrical capsule	gelatin	7.48 ± 0.05	21.40 ± 0.26	7.48 ± 0.05	36.355	
	B2	cylindrical capsule	gelatin	7.48 ± 0.05	21.39 ± 0.22	7.48 ± 0.05	36.34	
	C1	cylindrical capsule	gelatin	7.57 ± 0.02	21.62 ± 0.27	7.57 ± 0.02	36.75	
	C2	cylindrical capsule	gelatin	7.56 ± 0.02	21.69 ± 0.22	7.56 ± 0.02	36.81	
	D1	cylindrical capsule	gelatin	6.70 ± 0.05	20.74 ± 0.21	6.70 ± 0.05	34.125	
	D2	cylindrical capsule	gelatin	6.70 ± 0.03	20.85 ± 0.18	6.70 ± 0.03	34.25	
	E1	cylindrical capsule	pullulan	8.34 ± 0.05	23.52 ± 0.02	8.34 ± 0.05	40.185	
	E2	cylindrical capsule	pullulan	8.40 ± 0.06	23.55 ± 0.02	8.40 ± 0.06	40.34	
	F1	cylindrical capsule	gelatin	7.49 ± 0.05	21.69 ± 0.16	7.49 ± 0.05	36.66	
	G1	cylindrical capsule	HPMC ^2^	8.40 ± 0.07	23.13 ± 0.05	8.40 ± 0.07	39.92	
	H1	cylindrical capsule	HPMC ^2^	7.47 ± 0.08	21.22 ± 0.12	7.47 ± 0.08	36.15	
	I1	cylindrical capsule	cellulose	8.34 ± 0.05	23.06 ± 0.1	8.34 ± 0.05	39.725	
	J1	cylindrical capsule	HPMC ^2^	8.35 ± 0.04	23.31 ± 0.09	8.35 ± 0.04	40	
	K1	cylindrical capsule	HPMC ^2^	8.34 ± 0.05	23.39 ± 0.17	8.34 ± 0.05	40.055	
	L1	cylindrical capsule	cellulose	7.51 ± 0.03	21.02 ± 0.14	7.51 ± 0.03	36.025	
TABLETS	M1	arched circular	uncoated	11.19 ± 0.03	11.19 ± 0.03	5.80 ± 0.05	28.17	
	M2	arched circular	uncoated	11.19 ± 0.02	11.19 ± 0.02	5.77 ± 0.03	28.14	
	N1	flat circular	uncoated	12.12 ± 0.03	12.12 ± 0.03	4.84 ± 0.02	29.075	
	N1	flat circular	uncoated	12.11 ± 0.02	12.11 ± 0.02	4.83 ± 0.02	29.05	
	O1	arched oblong	uncoated	9.19 ± 0.02	19.09 ± 0.02	7.17 ± 0.08	35.435	acceptable
	O2	arched oblong	uncoated	9.19 ± 0.02	19.09 ± 0.02	7.15 ± 0.08	35.425	
	P1	arched circular	uncoated	9.13 ± 0.02	9.13 ± 0.02	4.71 ± 0.13	22.965	
	Q1	arched oval	uncoated	8.60 ± 0.01	17.17 ± 0.03	6.01 ± 0.02	31.775	
	R1	flat circular	uncoated	12.09 ± 0.02	12.09 ± 0.02	4.78 ± 0.02	28.95	
	S1	arched oblong	uncoated	9.66 ± 0.02	18.46 ± 0.03	6.47 ± 0.07	34.585	
	T1	arched circular	uncoated	9.15 ± 0.01	9.15 ± 0.01	4.80 ± 4.8	23.09	
	U1	arched oval	uncoated	9.92 ± 0.03	22.91 ± 0.04	8.45 ± 0.05	41.275	unacceptable
	W1	arched circular	uncoated	9.14 ± 0.02	9.14 ± 0.02	4.69 ± 0.06	22.97	acceptable
	Y1	arched circular	Glaze ^3^	11.17 ± 0.07	11.17 ± 0.07	6.48 ± 0.03	28.805	

^1^ shield material for capsules; coated/uncoated tablets. ^2^ HPMC—hydroxypropyl methylcellulose. ^3^ glaze consisted of HPMC, glycerin, carnauba wax. ^4^ a sum of three dimensions (width, length and depth) was calculated for each product. ^5^ FDA recommends that any dimension of tablet should not exceed 22 mm and the greatest acceptable capsule size is 00 (23.3 mm × 8.56 mm).

## Data Availability

All data is contained within the article and Supplementary Materials.

## Supplementary Materials

The following are available online at https://www.mdpi.com/article/10.3390/foods10081693/s1, Table S1: The producers’ information contained on the supplement label; Table S2: Iron and zinc content in dietary supplements. Figure S1: A diagram illustrating the experimental design.
