# Supplementary material for: Evaluation of Physicochemical Properties of Beetroot-Based Dietary Supplements
Source: Foods. 2021 Jul 22;10(8):1693. doi: 10.3390/foods10081693 (PMC8393586; doi:10.3390/foods10081693)
Supplement: Supplementary file 1 [file foods-10-01693-s001.zip › foods-1255523-supplementary.pdf]

Table S1. The producers' information contained on the supplement label

Form	Code	Number of dosage units	Product net weight (g)	The content of beetroot extract or preserves/dosage unit	Declared weight of the dosage unit (g)	Recommen-dation (dosage units/day)	Origin country
CAPSULES	A1	90	45	400 mg of root extract; 40 mg of nitrates	0.5	1x1 caps.	Poland (PL)
	A2						Poland (PL)
	B1	90	45	400 mg root extract (15:1); gelatine	0.5	1x1 caps.	Poland (PL)
	B2						Poland (PL)
	C1	60	35.76	dried juice concentrate; 38 mg of vitamin C; 2.8 mg of iron; capsule shell (gelatin of animal origin)	0.596	2x1 caps. during meal	Poland (PL)
	C2						Poland (PL)
	D1	30	11.3	268 mg of beetroot concentrate; vitamin C 20 mg ; iron gluconate 12 mg (1.4 mg iron); starch; anti-caking agent: magnesium salts of fatty acids; silicon dioxide	0.376	1x3 caps.	Poland (PL)
	D2						Poland (PL)
	E1	60	41.4	550 mg of *Beta vulgaris* extract 4:1; pullulan capsule	0.69	1x2 caps.	Poland (PL)
	E2						Poland (PL)
	F1	100	NA*	500 mg of beetroot; magnesium stearate; gelatin capsule	NA*	2-3x2 caps.	USA
	G1	60	NA*	700 mg of organic prepared beetroot (beetroot extract, maltodextrin) corresponding to 4620 mg of dried beetroot); vegetable capsule shell (hydroxypropylmethylcellulose)	NA*	1x2 caps. during meal	United Kingdom (UK)
	H1	100	NA*	500 mg of beetroot; vegetable capsules (modified cellulose); cellulose; silica; magnesium stearate	NA*	3x2 caps. during meal	USA
	I1	90	NA*	500 mg of beetroot extract (*Beta vulgaris*) (standardized for 0.3% betanin); cellulosan; silicon dioxide; vegetable fatty; vegetable mineral salts	NA*	3x1 caps. during meal	USA
CAPSULES	J1	60	NA*	450 mg of beetroot extract; bulking agent: microcrystalline cellulose; shells: hydroxypropyl methylcellulose	NA*	1x1-2 caps.	USA
	K1	90	NA*	500 mg of beetroot extract (*Beta vulgaris*) (5:1); bulking agents: maltodextrin, microcrystalline cellulose; vegetable capsule shell: hydroxypropyl methylcellulose; anti-caking agents: silicon dioxide, vegetable magnesium stearate	NA*	1x1 caps.	USA
	L1	60	NA*	300 mg of freeze-dried juice from organic pickled beetroot; micronized apple fiber; cellulose capsule shell	0.3	2x1 caps. before meal	Poland (PL)

	M1	60	33	500 mg of dried juice concentrate (refers to 2.75 g fresh beetroot); 1 mg of B_6; 1.25µg of B_{12}; bulking agent: microcrystalline cellulose; anti-caking agents: fatty magnesium salts, silicon dioxide	0.55	1-2x3 caps.	Poland (PL)
	M2						Poland (PL)
	N1	60	39	488 mg of beetroot concentrate; vitamin C 20 mg; iron gluconate 12 mg (1.4 mg iron); starch; anti-caking agent: magnesium salts of fatty acids; silicon dioxide	0.65	1x3	Poland (PL)
	N2						Poland (PL)
TABLETS	O1	120	111	500 mg of dried juice (refers to 3.5 g of fresh beetroot); anti-caking agent: magnesium salts of fatty acids; silicon dioxide	0.925	1-2x3 caps. during a meal or after a meal	Poland (PL)
	O2						Poland (PL)
	P1	100	35	132.375 mg of dicalcium phosphate; 132.375 mg of microcrystalline cellulose; 80 mg of beetroot extract (including 1% betaine); 2.25 mg vegetable magnesium stearate	0.35	1x2 tabl.	United Kingdom (UK)
	Q1	60	37.8	500 mg of fresh beetroot; maltodextrin; 40 mg of L-ascorbic acid (vitamin C); 7 mg iron II fumarate (iron); bulking agent: sorbitols; anti-caking agents: magnesium salts of fatty acids, silicon dioxide	0.63	1-2x1 tabl.	Poland (PL)
	R1	60	39	beetroot concentrate 500 mg; vitamin C 20 mg; 12 mg iron (II) gluconate (1.4 mg iron); starch; anti-caking agent: magnesium salts of fatty acids, silicon dioxide	0.65	3x1tabl.	Poland (PL)
	S1	60	NA*	500 mg of dried red beetroot concentrate; 1 mg vitamin B_6; 1.25 mg vitamin B_{12}; anti-caking agents: magnesium salts of fatty acids, silicon dioxide	NA*	3x1-2 tabl.	Poland (PL)
	T1	120	42	350 mg of beetroot extract 20:1 (80 mg of betanins); binder: dicalcium phosphate; emulsifier: microcrystalline cellulose; stabilizer: magnesium salts of fatty acids	0.35	1x1-2 tabl. during meal	United Kingdom (UK)
TABLETS	U1	100	146	1000 mg of beetroot extract; bulking agent: microcrystalline cellulose; anti-caking agent: stearic acid, magnesium stearate; stabilizer and solubiliser: sodium croscarmellose	1.46	1-3x1 tabl.	USA
	W1	120	42	160 mg of 10:1 beetroot extract (provides 1% nitrate); bulking agents: calcium hydrogen phosphate and cellulose; anti-caking agent: magnesium salts of fatty acids and silicon dioxide	0.35	1x1 tabl.	United Kingdom (UK)
	Y1	90	NA*	300 mg of beetroot extract; bulking agents: dicalcium phosphate, microcrystalline cellulose; anti-caking agents: stearic acid, silicon dioxide, magnesium stearate; glazing agents: hydroxypropyl methylcellulose, glycerin, carnauba wax	NA*	3x1 tabl.	United Kingdom (UK)

*NA - no data on the label provided from the manufacturer

Table S2. Analysis of iron and zinc content in dietary supplements

Form	Product	Average iron content		Declared added iron compound	Declared iron (elemental) content (mg/dosage unit)	% of producers declaration	Recom-menda-tion (dosage units/day)	Realization of RDA (%)		Average zinc content		Realization of RDA (%)		Zn:Fe ratio
		(mg/g)	(mg/ dosage unit)					women; 18 mg	men; 10 mg	(mg/g)	(mg/ dosage unit)	women; 8 mg	men; 11 mg	
CAPSULES	A1	0.016	0.006	*NA	*NA	*ND	1	0.04	0.06	0.002	0.001	0.01	0.01	1:6
	A2	0.031	0.012	*NA	*NA	*ND	1	0.07	0.12	0.002	0.001	0.01	0.01	1:12
	B1	0.014	0.006	*NA	*NA	*ND	1	0.03	0.06	0.002	0.001	0.01	0.01	1:6
	B2	0.035	0.014	*NA	*NA	*ND	1	0.08	0.14	0.004	0.001	0.02	0.01	1:14
	C1	5.41	2.777	iron(II) gluconate	2.8	99.17	2	30.85	55.54	0.007	0.004	0.10	0.07	1:694
	C2	6.75	3.394		2.8	121.22	2	37.71	67.88	0.008	0.004	0.09	0.07	1:848
	D1	3.89	1.214		1.4	86.69	3	20.23	36.42	0.008	0.003	0.09	0.07	2:809
	D2	5.86	1.772		1.4	126.60	3	29.54	53.16	0.008	0.004	0.14	0.10	1:443
	E1	0.016	0.007	*NA	*NA	*ND	2	0.08	0.14	<LOQ	<LOQ	0.00	0.00	*ND
	E2	0.012	0.006	*NA	*NA	*ND	2	0.06	0.12	<LOQ	<LOQ	0.00	0.00	*ND
	F1	0.001	0.003	*NA	*NA	*ND	3	0.06	0.09	<LOQ	<LOQ	0.00	0.00	*ND
	G1	<LOQ	<LOQ	*NA	*NA	*ND	2	0.0	0.00	0.010	0.007	0.18	0.13	*ND
	H1	<LOQ	<LOQ	*NA	*NA	*ND	6	0.0	0.00	0.014	0.008	0.58	0.42	*ND
	I1	<LOQ	<LOQ	*NA	*NA	*ND	3	0.0	0.00	0.001	0.001	0.03	0.02	*ND
	J1	<LOQ	<LOQ	*NA	*NA	*ND	2	0.0	0.00	<LOQ	<LOQ	0.00	0.00	*ND
	K1	<LOQ	<LOQ	*NA	*NA	*ND	1	0.0	0.00	0.007	0.004	0.06	0.04	*ND
	L1	0.017	0.015	*NA	*NA	*ND	2	0.17	0.30	0.032	0.009	0.23	0.17	3:5
TABLETS	M1	0.012	0.007	*NA	*NA	*ND	6	0.22	0.42	0.003	0.005	0.40	0.29	5:7
	M2	0.015	0.008	*NA	*NA	*ND	6	0.28	0.48	0.007	0.004	0.28	0.20	1:2

N1	1.77	1.189	iron(II) gluconate	1.4	84.91	3	19.81	35.67	0.008	0.005	0.19	0.14	1:238
N1	2.99	1.970		1.4	140.69	3	32.83	59.10	0.007	0.004	0.16	0.12	2:985
O1	0.039	0.040	*NA	*NA	*ND	6	1.33	2.40	0.003	0.004	0.29	0.21	1:10
O2	0.039	0.040	*NA	*NA	*ND	6	1.34	2.40	0.002	0.003	0.20	0.15	3:40
P1	0.006	0.007	*NA	*NA	*ND	2	0.08	0.14	0.014	0.005	0.13	0.09	5:7
Q1	3.83	8.960	iron(II) fumarate	7.0	128.01	2	99.56	179.20	0.004	0.003	0.06	0.05	*ND
R1	0.639	1.378	iron(II) gluconate	1.4	98.46	3	22.97	41.34	0.007	0.005	0.18	0.13	2:551
S1	0.001	0.005	*NA	*NA	*ND	6	0.16	0.30	0.001	0.001	0.11	0.08	1:5
T1	0.0013	0.015	*NA	*NA	*ND	2	0.17	0.30	0.002	0.001	0.02	0.02	1:15
U1	<LOQ	<LOQ	*NA	*NA	*ND	3	0.00	0.00	<LOQ	<LOQ	0.00	0.00	*ND
W1	0.004	0.005	*NA	*NA	*ND	1	0.03	0.05	0.002	0.001	0.01	0.01	1:5
Y1	0.003	0.005	*NA	*NA	*ND	6	0.17	0.30	0.003	0.002	0.09	0.07	2:5

Supplements enriched with iron compounds are highlighted in gray in the table.
*ND - not available; value cannot be calculated due to lack of data
*NA - no data from the manufacturer
LOQ $_{Fe}$ = 0.158 μg/mL; LOQ $_{Zn}$ = 0.03 μg/mL;

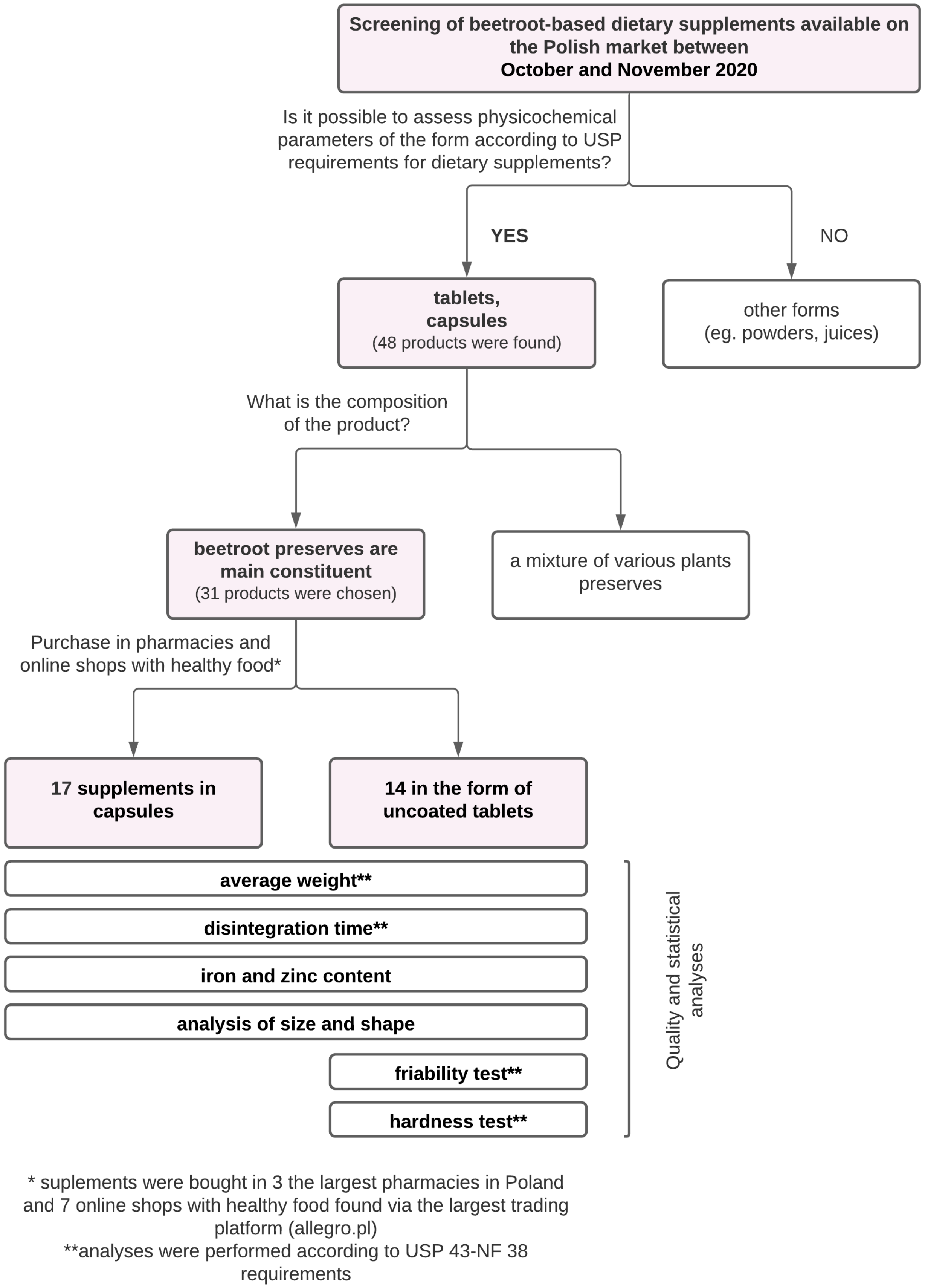

Figure S1: A diagram illustrating the experimental design
